# Patient-Reported Outcomes in Older Adults With Transthyretin Amyloid Cardiomyopathy: When the Heart Isn’t the Only Matter

**DOI:** 10.1016/j.jacadv.2025.102204

**Published:** 2025-10-03

**Authors:** Tessa Zeis, Vincent Chen, W.H.Wilson Tang, Parag Goyal, Mazen Hanna, Abdulla Damluji, Trejeeve Martyn

**Affiliations:** a Heart, Vascular & Thoracic Institute, Cleveland Clinic, Cleveland, Ohio, USA; b Amyloidosis Center, Cleveland Clinic Foundation, Cleveland, Ohio, USA; c Department of Medicine, Weill Cornell Medicine, New York, New York, USA; d Cardiovascular Center on Aging, Cleveland Clinic Foundation, Cleveland, Ohio, USA

**Keywords:** health-related quality of life, patient-reported outcomes, transthyretin amyloid

Transthyretin amyloid cardiomyopathy (ATTR-CM) is a progressive, infiltrative disease characterized by misfolded transthyretin proteins within the cardiac muscle that can influence the patient’s health-related quality of life (HR-QoL). As ATTR-CM has been diagnosed more frequently with more targeted treatments available, patient-reported outcomes (PROs) have been utilized to assess the trajectory of ATTR-CM both in routine clinical care and in ATTR therapeutic trials. Although several HR-QoL surveys are well-validated in heart failure (HF) syndrome more broadly, their applicability to ATTR-CM is less well understood.^1^ The deposition of transthyretin within the cardiac muscle, nervous system, soft tissues, ligaments/tendons, and joints leads to multisystem manifestations of disease. Common orthopedic histories in ATTR patients include spinal stenosis, bilateral carpel tunnel syndrome, biceps tendon rupture, and ocular findings such as periorbital purpura or vitreous opacities are often observed. Variant-related ATTR-CM with mixed phenotypes commonly presents with varying severity of small fiber peripheral and/or autonomic neuropathy causing numbness/tingling in the extremities, erectile dysfunction, diarrhea, and significant orthostatic symptoms.^2^ Given these broad manifestations of disease, traditional HF PROs may not differentiate impairments specific to ATTR-CM from broader HF syndrome complaints like shortness of breath. There may be additive value in evaluating, recognizing, and managing these multisystem symptoms when treating patients with ATTR-CM. Several recent PROs have focused on the multisystem aspects of ATTR but with limited utilization in clinical trials or real-world clinical settings. With longer-term follow-up due to progressively earlier diagnosis and better disease management, multidomain assessments can help clinicians and investigators understand disease progression and evaluate therapeutic strategies. With three Food and Drug Administration (FDA)-approved therapeutics and ongoing phase 3 studies testing RNA silencing, gene editing, and antibody fibril removal therapies, clinicians and patients will be searching for ways to differentiate therapies more quickly than long-term cardiovascular outcomes trials can offer. Given cross trial comparisons are problematic and there are few active comparator trials comparing ATTR-targeted therapies on the horizon, PROs may offer an avenue to understand the impact of different therapies in the shorter term. The correlation of prognostication and HF events with PROs does not signify the only metric for “success” rather, patient-centered measures of HR-QoL including mobility, fatigue, and independence could provide different or earlier signals of disease progression or regression. We aim to evaluate the limitations of commonly used HF PROs, strengths and weaknesses of ATTR-specific questionnaires, and discuss barriers of implementing these survey instruments in clinical/research settings.

## Patient-reported outcomes in ATTR-CM

PROs have gained increasing attention as key endpoints that can contribute to regulatory approval both in pivotal ATTR-CM studies and in HF trials more broadly. HR-QoL represents a patient-centered measure of disease burden. In a survey of patients with HF, 61% attached more weight to QoL over longevity; 9% and 14% reported they would trade 6 or 12 months of life for improved health and quality of life.^3^ PROs can provide a more holistic evaluation of how treatments and procedures may impact the patient as an individual and can help improve shared decision-making, treatment escalation, and the assessment of treatment response.^4^ A recent analysis comparing the HF validated Kansas City Cardiomyopathy Questionnaire (KCCQ) and Minnesota Living with Heart Failure Questionnaire (MLHFQ), and the nondisease specific Euro-Qol 5 dimension-5 levels (EQ-5D-5 L) in ATTR-CM found EQ-5D-5 L (includes Visual Analog Scale) and MLHFQ were predictors of all-cause mortality independent of biomarkers and all 3 questionnaires were predictive of combined all-cause mortality and HF-related hospitalization after adjustment for biomarkers.^5^ However, there have been notable examples wherein patient-reported improvement is uncoupled from improved clinical outcome, such as inotropes in HF, which improves QoL without improving prognosis, and alternatively beta-blockers, which have been shown to improve HF prognosis but may worsen QoL acutely.^6^^,^^7^ Therefore, the aim of PROs should be to capture the full patient experience living with the disease. Cardiovascular outcomes, like all-cause mortality, cardiovascular mortality, and HF hospitalization, have strong precedent in driving approval by the FDA but typically require large numbers of patients and long periods of follow-up to accrue sufficient events. However, there is a shift in FDA approval, such as with tricuspid transcatheter edge-to-edge repair, which was approved mainly on the basis of PRO and HR-QoL highlighting a recognition that symptom improvement and functional status, demonstrated by substantial and durable improvements in KCCQ score and NYHA functional class, are important to patients and clinically significant.^8^ In light of three FDA-approved therapies making placebo-controlled trials unethical and progressively “less sick” populations enrolled in ATTR-CM trials over time, CV outcome trials in ATTR-CM will be increasingly challenging and costly. In this context, recent ATTR-CM trials have favored composite endpoints or “win” ratio approaches that include functional endpoints like 6-minute walk distance, patient-reported outcome measures like KCCQ, used in combination with traditional CV outcomes like CV mortality, HF hospitalization, and worsening HF events.^9^ CV morbidity should not be the only lens to view disease progression in a complex syndrome.

PROs not only help to understand the total patient experience but also may be used to follow treatment effect, monitor for decline, or compare different treatments in ATTR-CM. Though caution should be exercised when directly comparing outcomes between trials, it is instructive to see consistent slowing in the decline of HR-QoL in ATTR-CM trials. In the ATTR-ACT trial, tafamidis was observed to slow the decline of all components of KCCQ scores with a mean difference in change of 13 with over 40% of patients treated with tafamidis having no change or an improvement in KCCQ scores at 30 months.^10^ During an extension study that continued follow-up through 60 months, the reduction in the decline of HR-QoL effect of tafamidis was still seen.^11^ Acoramidis led to improved quality of life assessed by KCCQ as well over 30 months with a mean difference in change of 9.94 points.^9^ The recent vutrisiran in patients with transthyretin amyloidosis trial showed vutrisiran resulted in less decline of KCCQ score with least squares mean difference of 5.8 points.^12^ Importantly, the baseline characteristics of these trials are markedly different with the placebo arm of Helios-B (vutisaran vs placebo) with better all-cause survival than the treatment arm of ATTR-ACT (tafamidis vs placebo).^13^

## Multisystem assessment in older adults

As we now understand ATTR-CM to be a multisystem disease affecting multiple domains of life, there have been recent developments of ATTR-specific PROs. However, to date, the most utilized PROs in trials, as seen above, are the KCCQ and MLHFQ, which have been mostly validated in broader, nonamyloid, HF populations.^14^ These questionnaires include an evaluation of cardiac symptoms as well as other domains like psychological impact or limitations in activities of daily living (ADLs). Other questionnaires evaluating HR-QoL in ATTR-CM include the EQ-5D-5 L/EQ-Visual Analog Scale, and 36-item short form survey, which are nondisease-specific questionnaires evaluating nonspecific symptoms and other important domains like emotional impact, ADLs, mobility, and others. When deployed in ATTR-CM patients, these commonly used questionnaires may fall short of understanding the multisystem impact of or symptoms more specific to ATTR-CM. A neuropathy-specific questionnaire initially validated for diabetic neuropathy but has been utilized for ATTR-PN to evaluate PROs in various trials involving RNA silencer therapy is the Norfolk Quality of Life-Diabetic Neuropathy questionnaire though it does not evaluate cardiac symptoms.^15^ A list of notable PROs and their domains that have been utilized for ATTR-CM previously are described in Table 1.

**Table 1:** Comparison of HR-QL Instruments Utilized for ATTR-CM

	Items	Minimum Score	Maximum Score	Domains/Dimensions	Additional Notes
Kansas City Cardiomyopathy Questionnaire (KCCQ-23)	23	0	100	• Symptom frequency • Physical limitation • Quality of life • Social limitation	• Lower is more limiting • Raw score from Likert scales, then converted to a scaled score from 0 to 100 • Heart failure specific
Minnesota Living with HF Questionnaire (MLHFQ)	21	0	105	• Physical • Emotional	• Higher is more limiting • Raw score from Likert scales, 0-5 for each item • Mostly HF specific but includes questions on general function
Euro-Qol 5-Dimension, 3-level (EQ-5D-3 L), Index score	5	0	1	• Mobility • Self-care • Daily activities • Pain/discomfort • Anxiety/depression	• Lower is more limiting • Raw score from Likert scales, 1-3 for each item, then converted by a proprietary regional-weighting formula to an index score where 1 is the best health status and 0 (or less) is the worst health status • Generic illness
Euro-Qol Visual Analog Scale (EQ-VAS)	1	0	100	• Overall health	• Lower is more limiting • Patient marks a single location on a numbered scale • Generic illness
36-Item Short Form Survey (SF-36)	36	0	100	• Limitations in physical activities, social activities, usual role activities due to physical or emotional problems • Pain • Mental health • Energy • General health perception	• Lower is more limiting. • Each item score 0-100 range, then items are averaged together for each domain. Each domain then gets a separate score from 0 to 100% • Generic illness
Norfolk Quality of Life-Diabetic Neuropathy (Norfolk QOL-DN)	35	−4	136	• Large fiber neuropathy • Small fiber neuropathy • Autonomic neuropathy • Daily activities • Symptom burden	• Higher is more limiting • Validated for familial amyloid polyneuropathy • Includes questions on general function
Amylo-AFFECT-QOL	33	0	99	• HF • Vascular dysautonomia • Neuropathy • Gastrointestinal and urinary dysautonomia • Skin or mucosal involvement	• Higher is more limiting • Raw score from Likert scales, 0-3 for each item • Specific to cardiac amyloid (ATTR and amyloid light chain) • Novel, not much data yet
ATTR-QOL	37	0	100	• Cardiac • Neuropathic (peripheral) • Neuropathic (autonomic) • Other (skin, vision, memory) • Physical limitations • Social impact • Mental/emotional impact	• Higher is more limiting • 0-5 for each item, except yes/no for item about working, complete score derived by averaging item scores within each scale. • Optional items can capture type of ATTR and self-reported past diagnosis of 11 separate comorbid conditions • Specific to ATTR
ITALY	30	0	100	• Physical limitation • Symptom stability • Symptom frequency • Symptom burden • Self-efficacy • QoL	Lower is more limiting 0-5 for each item 2 separate questionnaires for wt vs variant-ATTR Scores were sum of all scores divided by maximum score to all questions

QoL = quality of life; ATTR = transthyretin Amyloid; HF = heart failure; Amylo-AFFECT-QOL = Amyloidosis Affected Health-Related Quality of Life Questionnaire; ITALY = Impact of Transthyretin Amyloidosis in Life qualitY.

## Transthyretin-specific PROs

A multisystem questionnaire specific to cardiac amyloid (both ATTR and amyloid light chain) has been developed and validated, Amyloidosis Affected Health-Related Quality of Life Questionnaire (Amylo-AFFECT-QOL), which is an amyloid-specific HR-QoL questionnaire created to help understand the breadth of disease impact. The Amylo-AFFECT-QOL questionnaire captures the multi-organ symptomatology including HF, neuropathy, gastrointestinal, genitourinary, and vascular dysautonomia and skin complications in amyloid and was found to have both diagnostic and prognostic value.^16^ This questionnaire, however, only evaluates physical symptoms and misses the other aspects of patients’ lives that affect their QoL. ATTR-CM is mainly a disease affecting older adults who typically have multiple chronic conditions that compound the effect ATTR has on quality of life, symptoms, and overall functional capacity. These patients struggle with polypharmacy, disability, social isolation, frailty, sarcopenia, and mental health disorders that contribute to overall morbidity.^17^ Given the potential complexities of a multisystem disease, geriatric population, and HF syndrome, comprehensiveness and practicality of administration should be balanced for a survey to be used broadly.

A newer PRO that is ATTR-CM specific but also includes a broader assessment of social, physical, and emotional limitations caused by the disease in addition to symptoms specific to the disease is the ATTR-QOL questionnaire. Strong correlations of the ATTR-QOL Impact scores and related domains of Short Form- 36 (SF-36), KCCQ-12, and Norfolk Quality of Life-Diabetic Neuropathy were found on validation study.^18^ This questionnaire is meant to be used for both wild-type-ATTR and variant-ATTR.

Even more specific to the type of ATTR, the ITALY (Impact of Transthyretin Amyloidosis in Life qualitY) questionnaires were created specifically for wt-ATTR and v-ATTR. This includes 2 separate questionnaires for wt-ATTR and v-ATTR given the differences in symptomatology between these. In an evaluation of their correlation with overall KCCQ and SF-36 scores, the wt-ATTR ITALY score showed close correlation with these scores, while the v-ATTR displayed weak correlations with the overall KCCQ and SF-36 scores, an important observation that was attributed to the heterogeneity in clinical presentation of v-ATTR.^19^

## Future outlook

The development of PROs specific to ATTR-CM has been blossoming in recent literature, including Amylo-AFFECT, ATTR-QOL, and ITALY questionnaires. This move to a more disease-specific PRO is important to accurately capture the total patient experience or impairment of ATTR as mentioned previously. While these advances are promising, such novel instruments have not been evaluated in clinical trials. More validation studies are likely needed prior to widespread use of any of these measures. A limitation of the Amylo-AFFECT questionnaire is that it only assesses symptoms and does not query other patient-relevant domains. The ITALY separate questionnaires for wt vs v-ATTR may not be necessary as we are finding more overlap in symptoms.^18^ All of these questionnaires are quite extensive, which may be reasonable in a trial setting but may be cumbersome within the workflow of busy outpatient clinics, where patient questionnaire fatigue and electronic health portal integration are ongoing challenges.

Moving forward, there is room for broader deployment of ATTR-specific PROs that capture the total patient experience and impairment associated with ATTR-CM. Prognostication is only one domain in which to evaluate a PRO particularly given national amyloidosis center staging shows ongoing prognostic value. We believe the additive value of an ATTR-specific questionnaire includes an emotional assessment, social/ADL limitations, and overall QOL assessment that is important for the primarily older population affected by ATTR. PROs are an important instrument in assessing clinical progression and outcomes in a rapidly evolving diagnostic and therapeutic ATTR-CM landscape.

## Funding support and author disclosures

Dr Tang has served as a consultant for Cardiol Therapeutics, Genomics plc, Zehna Therapeutics, WhiteSwell, 10.13039/100008497Boston Scientific, CardiaTec Biosciences, Intellia Therapeutics, Bristol Myers Squibb, Alleviant Medical, 10.13039/100006396Alexion Pharmaceuticals, Salubris Biotherapeutics, BioCardiac Inc; and has received honorarium from Springer, Belvoir Media Group, and American Board of Internal Medicine. Dr Hanna has served on Advisory Boards for 10.13039/100006400Alnylam, Eidos, Ionis, 10.13039/100006396Alexion pharmaceuticals, and Pfizer. Dr Martyn has served as an advisor or has received consulting fees from Prolaio, 10.13039/100001003Boehringer Ingelheim/Eli Lilly, BridgeBio, NovoNordisk, Cleveland Clinic/American Well Joint Venture, BridgeBio, 10.13039/100004319Pfizer, Apricity Robotics, and Kilele health and has received grant support from Ionis Therapeutics, Fire1, AstraZeneca, and the 10.13039/100006795Heart Failure Society of America (HFSA). All other authors have reported that they have no relationships relevant to the contents of this paper to disclose.
